# Supplementary material for: Human-sized magnetic particle imaging for brain applications
Source: Nat Commun. 2019 Apr 26;10:1936. doi: 10.1038/s41467-019-09704-x (PMC6486595; doi:10.1038/s41467-019-09704-x)
Supplement: Supplementary file 2 — Description of Additional Supplementary Files [file 41467_2019_9704_MOESM2_ESM.docx]

**Description of Additional Supplementary Files**

**File Name: Supplementary Video 1**

**Description:** Video of the dynamic control experiment with high dose (850 µg Fe). Video and reconstruction were synchronized by hand to the arrival of the bolus. The bolus was injected as fast as possible in approximately 1.2 m distance of the scanner. One can see that the bolus arrives in the left tube slightly before the right tube which is visible in the reconstructed image series as well.

**File Name: Supplementary Video 2**

**Description:** Video of the dynamic stenosis experiment with high dose (850 µg Fe). Video and reconstruction were synchronized by hand to the arrival of the bolus. The bolus was injected as fast as possible in approximately 1.2 m distance of the scanner. The left feeding hose was pinched suppressing any flow within the phantom. As a result the perfusion deficit is visible in the reconstructed image series.

**File Name: Supplementary Video 3**

**Description:** Video of the dynamic control experiment with low dose (85 µg Fe). Video and reconstruction were synchronized by hand to the arrival of the bolus. The bolus was injected as fast as possible in approximately 1.2 distance of the scanner. The spatial resolution was less which could be explained by the low dose, however the two tubes could still be separated.

**File Name: Supplementary Video 4**

**Description:** Video of the dynamic stenosis experiment with low dose (85 µg Fe). Video and reconstruction were synchronized by hand to the arrival of the bolus. The bolus was injected as fast as possible in approximately 1.2 m distance of the scanner. The left feeding hose was pinched suppressing any flow within the phantom. As a result the perfusion deficit is visible in the reconstructed image series.
